# Functional Neuroplasticity of Adults with Partial or Complete Denture Rehabilitation with or without Implants: Evidence from fMRI Studies

**DOI:** 10.3390/nu15071577

**Published:** 2023-03-24

**Authors:** Andy Wai Kan Yeung, Wai Keung Leung

**Affiliations:** 1 Oral and Maxillofacial Radiology, Applied Oral Sciences and Community Dental Care, Faculty of Dentistry, The University of Hong Kong, Hong Kong SAR, China; 2 Periodontology and Implant Dentistry, Faculty of Dentistry, The University of Hong Kong, Hong Kong SAR, China

**Keywords:** cognition, dental implant, dentistry, denture, fMRI, mastication, neuroplasticity, nutrition, oral rehabilitation, prosthodontics

## Abstract

Tooth loss may affect food ingestion and, consequently, nutrition intake. The neuroimaging literature using functional magnetic resonance imaging (fMRI) was reviewed to summarize the changes in brain functions in response to denture rehabilitation in patients with partial or complete edentulous dentition. Overall, this review covered nine fMRI studies on denture rehabilitation. Eight recruited complete edentulous patients, whereas one recruited partially edentulous patients. The risk-of-bias assessment revealed concerns regarding all nine studies. Due to the heterogeneity of the studies and the lack of brain coordinates reported, a meta-analysis could not be conducted, and this review could only summarize the findings without statistical validation. The evidence from jaw-clenching studies suggested that implant-supported fixed dentures could be the best option, as compared to implant-supported overdentures and complete dentures, as it was associated with higher brain activity levels in various brain regions, including those corresponding to the primary sensory (postcentral gyrus) and motor cortices (precentral gyrus). Gum-chewing studies indicated that perhaps the medial and middle frontal gyri were associated with food comminuting and food mixing, which could be improved by the full replacement of the dental arch, instead of only partial replacement. All the fMRI studies described the functional neuroplasticity of the patients undergoing denture rehabilitation and suggested that certain rehabilitation options were more beneficial in restoring masticatory functions, as well as their associated brain activity levels.

## 1. Introduction

Tooth loss can have a serious impact on food choice and food ingestion and, subsequently, the intake of adequate nutrients [1,2,3]. A recent meta-analysis of studies in adults aged 60 years and older concluded that patients with complete edentulism or non-functional dentition were 21% more likely to have symptoms related to malnutrition and malnourishment [4]. Tooth loss has multiple causes, with dental caries and periodontal disease being the most common [5,6]. Human studies have reported associations between tooth loss and altered brain structures. As compared to patients with at least 20 teeth, completely edentulous patients were found to have a significant atrophy of gray matter in the right hippocampus and caudate [7]. Among patients with cognitive impairments, an increased number of missing teeth (i.e., tooth loss) was associated with reduced gray matter volume in the bilateral primary motor cortex and premotor cortex, as well as the left hippocampus and parahippocampal region [8]. In an elderly cohort without cognitive impairments, the gray matter volume at the premotor cortex was found to be positively correlated with masticatory performance [9]. Meanwhile, a 9-year prospective population-based study on dementia-free adults aged 60 years and over reported that tooth loss was associated with a steeper cognitive decline, as well as lower total brain and gray matter volumes [10]. Separate from masticatory performance and cognitive performance, generalized gray matter volume reduction was found among people with narcolepsy, a sleeping disorder resulting from an orexin deficiency [11], which is a hormone that also enhances appetite [12]. However, it was reported that there was no significant difference in the brain responses to vibrotactile stimuli applied to a natural tooth or a dental implant [13].

Functional magnetic resonance imaging (fMRI) technology measures brain activity in a non-invasive manner by detecting the blood oxygen level-dependent (BOLD) signals at a high spatial resolution [14]. Many fMRI studies have been published that evaluated oral functions and eating-related conditions, such as dental pain [15], swallowing [16], and tasting [17,18]. In terms of tooth loss, a previous fMRI study showed that fully dentulous patients had higher brain activity levels in the primary somatosensory and primary motor cortices, thalamus, basal ganglia, and the insula cortex during teeth-tapping, as compared to fully edentulous patients with dentures that had replaced their missing teeth [19].

Though the aforementioned findings were insightful, it was more clinically important to assess whether denture rehabilitation in patients with partial or complete edentulous dentition could restore brain activity levels when patients then performed oral and/or cognitive tasks. This review covered existing evidence from relevant, published fMRI studies. For completeness, readers are also referred to a review of the respective non-fMRI studies (e.g., functional near infra-red spectroscopy, near infra-red optical topography, and electroencephalography) [20].

## 2. Materials and Methods

**Figure 1:**
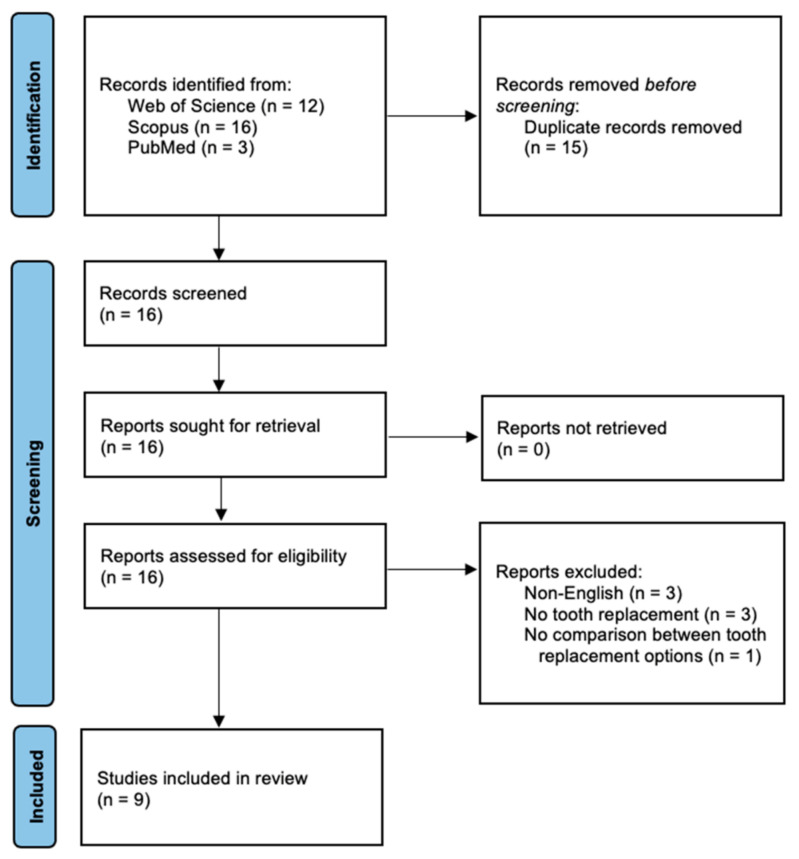
PRISMA 2000 flow diagram showing the literature screening process.

The Population, Intervention, Comparison, and Outcomes (PICO) considerations of this review were as follows. Population: Patients with missing teeth. Intervention: Tooth replacement by fixed or removable dentures. Comparison: Different tooth replacement methods. Outcomes: Brain activity levels in response to any tasks conducted by patients wearing different types of dentures. On 1 February 2023, 3 electronic literature databases were searched for published reports of fMRI studies on denture rehabilitation, namely the Web of Science, Scopus, and PubMed. The search string was: ((denture* OR overdenture*) AND (fMRI OR “functional magnetic resonance” OR “functional MRI”)). For the Web of Science and Scopus, the title, abstract, and keyword fields were searched, whereas for PubMed, the title and abstract fields were searched. No additional filters, such as publication language or document type, were applied. Initially, the results yielded a total of 31 papers (Figure 1). After removing 15 duplicate records, the remaining 16 papers were assessed for eligibility. These papers were subsequently excluded if they were review papers (*n* = 0), non-English publications (*n* = 3), involved no tooth replacement for their subjects (*n* = 3), or no comparison between different tooth replacement options (*n* = 1). Finally, nine studies remained. The cited and citing papers of these nine studies were also reviewed and confirmed so that no paper was overlooked.

Regarding the risk-of-bias assessment, a randomized crossover study was assessed by the Risk-of-Bias 2 (RoB 2) for crossover trials [21]. Non-randomized studies were assessed by risk-of-bias assessment tools for non-randomized studies (RoBANS) [22], which had been used by the authors in another systematic review on neuroimaging studies [18].

## 3. Results and Discussion

### 3.1. Overview of the Nine Included Studies

Table 1 shows a summary of the nine studies included in this review. The studies were conducted in Asia (i.e., China, India, and Japan) and Oceania (i.e., Australia). Each study recruited approximately 4–20 patients. One study reported details on the sample size calculation [23], whereas another study simply mentioned that the “sample size was calculated to be seven” [24]. No study explicitly mentioned the socioeconomic background or ethnicity of the subjects. Randomization of patients into two groups was performed by a single study [25]. The denture rehabilitation options involved in these nine studies were divided into three categories: complete dentures, implant-supported overdentures, and implant-supported fixed dentures. For complete denture treatment, all studies involved complete dentures for both arches. For implant-supported overdenture treatment, most studies involved a maxillary complete denture and a mandibular implant-supported overdenture. The mandibular implant-supported overdenture was supported by two implants in the bilateral canine-premolar region, as previously described, for good retention [26]. A single study recruited patients with implant-supported overdentures in both the maxillary and mandibular arches [27]. The majority of the studies conducted within-group comparisons, with the same group of patients receiving different denture treatments at different time points (longitudinal). Two studies were cross-sectional with between-group comparisons [27,28]. Meanwhile, only three studies collected brain activity data from their patients before the experimental interventions: Two collected data while patients were wearing their old complete dentures [23,29], and one collected data while patients were completely edentulous, without dentures [24]. Brain activation in response to several tasks was assessed, with the most popular task being jaw (denture teeth) clenching and gum-chewing. Denture teeth-tapping, lip-pursing, memory recall, continuous performance tasks, and go/no-go task were used in one study each.

**Figure 2:**
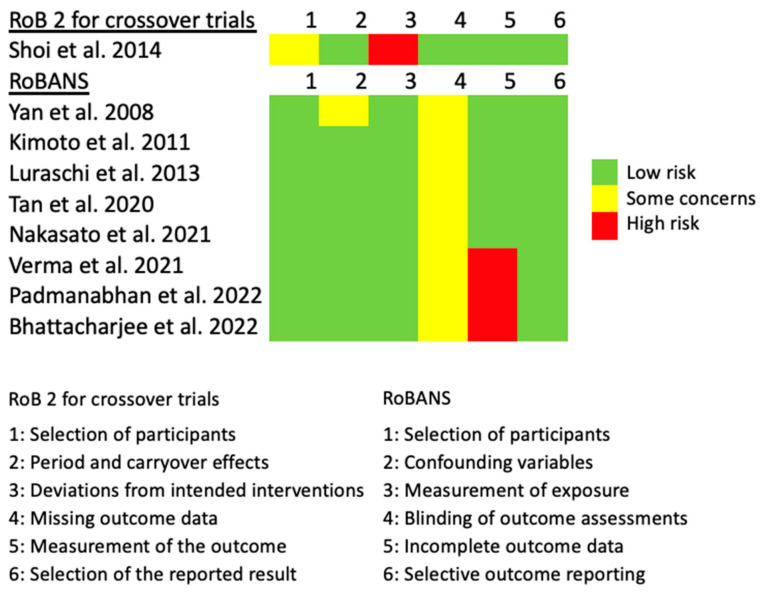
Risk-of-bias assessment of the studies. RoB 2, Risk-of-Bias 2 assessment tool [21]. RoBANS, risk-of-bias assessment tools for non-randomized studies [22]. Shoi et al. 2014 [25]; Yan et al. 2008 [27]; Kimoto et al. 2011 [30]; Luraschi et al. 2013 [29]; Tan et al. 2020 [31]; Nakasato et al. 2021 [23]; Verma et al. 2021 [32]; Padmanabhan et al. 2022 [24]; Bhattacharjee et al. 2022 [28].

The risk-of-bias assessment is summarized in Figure 2. For Shoi et al. [25], concerns were raised regarding the selection of the participants, as they had only mentioned that randomization had been performed, without details. Significant risks were identified in the intended interventions, as patients were aware of the assigned intervention and, therefore, not blinded regarding the randomization. For all non-randomized studies, concerns were raised regarding the blinding of outcome assessments, as none of them had declared whether the investigators who had analyzed the data had been blinded concerning patient group allocation. For Yan et al. [27], concerns were raised regarding confounding variables, as they had compared patients who already had undergone different types of denture rehabilitations, without disclosing or accounting for the differences in the socioeconomic status between the patient groups. Significant risks were identified in the incomplete outcome data for three studies [24,28,32], as they had analyzed the fMRI data based on their subjective judgments (i.e., “no signal”, “mild signal”, versus “strong signal”) instead of the routine statistical tests based on numerical values. Due to the heterogeneity of the studies and the lack of brain coordinates reported by the studies, a neuroimaging meta-analysis was not feasible. Therefore, a narrative summary is presented instead.

Circumstantial evidence from jaw-clenching studies regarding complete edentulous individuals rehabilitated with complete dentures, implant-supported overdentures, or implant-supported fixed dentures (*n* = 2) [27,28] suggested that the implant-supported fixed dentures could be the best option, as compared to implant-supported overdentures and complete dentures, as it was associated with higher brain activity levels in various brain regions, including those corresponding to the primary sensory (PostCG) and motor cortices (PreCG). Gum-chewing studies (*n* = 4, one with partial edentulous participants) [23,24,25,31] found that the brain activity levels in various brain regions, such as the prefrontal cortex, PreCG, and the parietal cortex, could be improved by having a dental arch fully replaced, instead of partially replaced, or having a new complete denture replacement. Implant-supported overdenture were associated with lower brain activity levels than those in edentulous patients, but the studies related to these conditions had mixed results, as compared to those with complete dentures.

### 3.2. Brain Functions of Rehabilitated Partial/Complete Edentulous Jaws during Various Tasks

#### 3.2.1. Jaw-Clenching

Jaw- or tooth-clenching is the action of holding the upper and lower teeth together tightly and forcefully. It is achieved by exerting a large bite force. Loss of teeth, especially the posterior teeth, naturally reduces the maximum bite force exerted during clenching. Luraschi et al. [29] recruited 11 complete denture wearers (6 males and 5 females, mean age ± SD: 71.4 ± 4.8 years, fully edentulous for 22.9 ± 13.4 years) and asked them to clench their denture teeth, tap their denture teeth fast in a constant rhythm, and purse their lips as if preparing for a kiss. Their brain activity levels while performing these tasks were recorded. As a longitudinal study, the subjects underwent these experimental procedures four times: (1) while were wearing their old dentures (T0), (2) on the day they received a new pair of dentures (T1), (3) one week after wearing new dentures (T2), and (4) three months after wearing new dentures (T3). The results showed the right and left precentral gyrus (PreCG) and the right and left postcentral gyrus (PostCG) were commonly activated across all three tasks of clenching, tapping, and lip-pursing. Subsequently, the activity levels of the PreCG and PostCG were compared among the four time points in a region-of-interest (ROI) analysis. The results showed that the activity level of the PreCG and PostCG for clenching had a significant increase at T2, as compared to T0, and then returned to baseline levels at T3. Meanwhile, there were no significant changes in their activity levels over time for teeth-tapping and lip-pursing. Separate from the brain activity data, the authors also evaluated chewing efficiency (assessed by measuring how well the participants could mix 2 pieces of chewing gum of different colors over 20 cycles of chewing) and maximum bite force. Interestingly, they found that the chewing efficiency significantly improved between each time point after receiving new dentures (i.e., from T1 to T2, and from T2 to T3), whereas the overall change from T0 (while wearing old dentures) to T3 was insignificant. The maximum bite force also significantly increased from T2 to T3, without an overall significant change from T0 to T3. These results suggested that cortical changes may have occurred in the PreCG and PostCG over the course of replacing their old complete dentures with new ones. After a possible habituation process of approximately three months, the participants had adapted to the new dentures and, thus, their brain activity levels returned to baseline. The PreCG and PostCG activity corresponded to the primary motor cortex and primary somatosensory cortex, respectively.

Meanwhile, Tan et al. [31] recruited 4 edentulous patients (2 males and 2 females, mean age ± SD: 73.0 ± 1.4 years, fully edentulous period not reported) and asked them to clench their teeth during an fMRI scan. The fMRI scan was performed thrice: three months after wearing new complete dentures (baseline), one week after switching to implant-supported overdentures, and six weeks after wearing implant-supported overdentures. The results showed that all sensory and motor-related brain regions showed reduced activity levels one week after switching to the implant-supported overdentures, but many returned to baseline levels six weeks after wearing implant-supported overdentures. Similarly, Luraschi et al. [29] found that the habituation process could take more than one month (from six weeks to three months). One difference between these two studies was that Luraschi et al. [29] had reported an initial increase in brain activation after switching from old complete dentures to new complete dentures, whereas Tan et al. [31] had reported an initial decrease in brain activation after switching from complete dentures to implant-supported overdentures.

Furthermore, Verma et al. [32] recruited 12 edentulous patients (sex not reported, mean age: 59.2 years, age range: 40–70 years, fully edentulous period not reported) and fabricated complete dentures for each participant. After the patients had worn the newly fabricated complete dentures for three months, they were instructed to clench their denture teeth inside the fMRI scanner in order to record their brain activity levels, as compared to their resting conditions. After the initial scan, they received two implant-supported mandibular overdentures “according to the standard Brånemark-system protocol”, and then they underwent the second scan after wearing these dentures for three months. It should be noted that the authors cited an irrelevant reference, [33] (Reference #6 in their reference list), to support the use of the standard Brånemark-system protocol. They may have intended to cite an earlier study, [30] (Reference #9 in their reference list). In this study, the authors examined the fMRI data and subjectively rated the data from 0 to 2: no signal, mild signal, and strong signal, respectively. The results showed that after wearing implant-supported mandibular overdentures for three months, the patients had significantly stronger signals in various brain regions, such as the PreCG, PostCG, hippocampus, insula, frontal lobe, and temporal lobe. The authors elaborated that since the hippocampus and the insula played roles in memory processing, the increased activity levels observed in these structures could imply beneficial effects on memory function against dementia by having implant-supported overdentures, rather than complete dentures. Readers, however, should be aware that the statistical method used in this study (a subjective rating on brain signal intensity) was rather uncommon, as it did not input the fMRI data into dedicated software to perform standard data analytics.

The following two studies reported inter-group differences. First, Bhattacharjee et al. [28] recruited 18 edentulous patients (10 males and 8 females, mean age ± SD: 60.7 ± 4.0 years, fully edentulous period not reported) and allocated them to 3 treatment groups: (1) complete dentures; (2) maxillary complete denture and mandibular implant-supported overdenture; and (3) implant-supported fixed dentures. FMRI scans were performed four weeks after treatment completion. The method to evaluate the brain activity levels followed the example of Verma et al. [32]. The results showed that patients wearing implant-supported fixed dentures had significantly more activation in the PreCG and PostCG, angular gyrus, and the temporal lobe. However, there were no group differences in terms of active tactile sensibilities, i.e., determining the presence/absence of articulating foil with various thicknesses. There were also no group differences in terms of stereognostic ability, i.e., identifying the correct shapes of objects placed in the mouth. Overall, the authors concluded that the implant-supported fixed dentures were the best option, as the peripheral feedback pathway appeared to be restored. Meanwhile, Yan et al. [27] recruited 20 edentulous patients (13 males and 7 females, mean age ± SD: 59.9 ± 7.4 years, fully edentulous period not reported) who had already received rehabilitative treatments (from 8 months up to 5 years) with either: (1) complete dentures; (2) a pair of implant-supported overdentures or maxillary complete denture with mandibular implant-supported overdenture; or (3) implant-supported fixed dentures. The results showed that a higher proportion of edentulous patients wearing implant-supported fixed dentures had brain activation in the precentral gyrus (PreCG) and postcentral gyrus (PostCG) during jaw-clenching, as compared to those wearing implant-supported overdentures and complete dentures [27]. The authors did not adjust for possible higher socioeconomic class of those who could afford fixed implant reconstructions.

**Figure 3:**
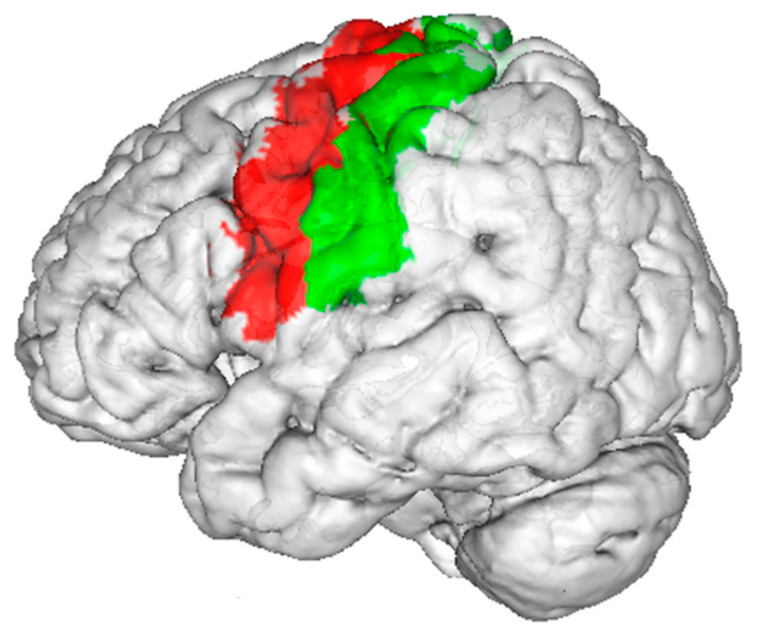
The precentral gyrus (red) and postcentral gyrus (green) had more activation during jaw-clenching with implant-supported fixed dentures than implant-supported overdentures and complete dentures.

To summarize, the evidence from the cross-sectional and/or retrospective studies suggested that the implant-supported fixed dentures appeared to be the best option, as compared to implant-supported overdentures and complete dentures, as this group of patients showed higher brain activity levels in various brain regions, including those corresponding to the primary sensory and motor cortices (Figure 3). The evidence from a longitudinal study also showed that patients had higher brain activity levels after wearing implant-supported overdentures, as compared to wearing complete dentures. Meanwhile, a longitudinal study showed that replacing old complete dentures with new ones could require a habituation process of about three months, after which the brain activity level in response to jaw-clenching would return to the pre-study levels. Readers should be aware that though there were multiple fMRI studies on jaw-clenching, all of them had, at most, 20 patients.

#### 3.2.2. Gum-Chewing

Kimoto et al. [30] recruited 4 edentulous patients (3 males and 1 woman, age range: 64–79 years, fully edentulous period not reported) and asked them to chew a stick of tasteless, odorless gum during 2 fMRI scans: the first performed after 1 month of receiving complete dentures; and the second 3 months later, when the patients received a maxillary complete denture and a mandibular implant-supported overdenture. The second scan was performed one month after receiving a new pair of dentures/overdentures. The authors reported that the bilateral prefrontal cortex had reduced activity levels when wearing the maxillary complete dentures with the mandibular implant-supported overdentures, as compared to complete dentures. It was postulated that repeated simple motor task could cause reduced activation in the respective motor regions [34]. Furthermore, Kimoto et al. [30] reasoned that complete dentures were usually more mobile (poorer stability) and induced a “more kinematically irregular and unstable chewing pattern”, as compared to implant-supported overdentures. In other words, implant-supported overdentures could lead to more regular, repeated simple motor actions of chewing and, hence, reduced activation.

Padmanabhan et al. [24] recruited 10 edentulous patients (4 males and 6 females, age range: 62–91 years, fully edentulous for at least 1 year) and asked them to chew a stick of gum during fMRI scans. It was not described if the chewing gum was tasteless and odorless. The fMRI scan was performed thrice: at T0 when they were edentulous, at T2 when they had worn complete dentures for 3 months, and at T3, when they had worn implant-supported overdentures for 3 months. The results showed that the activity levels in the prefrontal cortex were significantly reduced when the patients had worn implant-supported overdentures, as compared to T0 (edentulous). Meanwhile, the patients with implant-supported overdentures had significantly higher activity levels in the insula but lower levels in the hippocampus, as compared to when they had worn complete dentures. Regarding the reduced activity levels in those wearing implant-supported overdentures, as compared to complete dentures, the authors referred to the explanation by Kimoto et al. [30].

Nakasato et al. [23] recruited 14 edentulous patients (4 males and 10 females, mean age ± SD: 80.2 ± 5.9 years, wearing old complete dentures for at least 1 year) and asked them to chew a stick of tasteless, odorless gum during fMRI scans. The patients were scanned again three months after wearing new complete dentures. The results showed that wearing new dentures during gum-chewing had significantly higher brain activity levels in the PreCG, prefrontal cortex, putamen, cerebellum, and inferior parietal lobe, than wearing old dentures. It was also reported that the electromyography (EMG) signal of the masseter muscles was significantly higher when patients were wearing new dentures. Moreover, the EMG activity of the masseter muscles was positively correlated with the fMRI signal in numerous brain regions, such as the hippocampus, parahippocampal gyrus, and the PostCG. Unfortunately, the authors did not clarify if this correlation had been based on data obtained with old dentures, new dentures, or the differences between the old and new dentures. At the same time, the patients showed improved cognitive function after wearing new dentures, in terms of visual attention and memory recall, as assessed by neuropsychological tests (Trail-Making Test Part A, TMT-A; Rey Auditory Verbal Learning Test, RAVLT; and Rey–Osterrieth Complex Figure Test, R-OCFT). The authors concluded that by wearing complete dentures of improved quality (new versus old), the improved masseter muscle function could increase brain activity levels in response to chewing and could be beneficial for attention and memory functions. However, it should be noted that the authors did not report any statistical relationships between the cognitive functions (TMT-A, RAVLT, R-OCFT) and masseter muscle functions/fMRI data related to gum-chewing.

**Figure 4:**
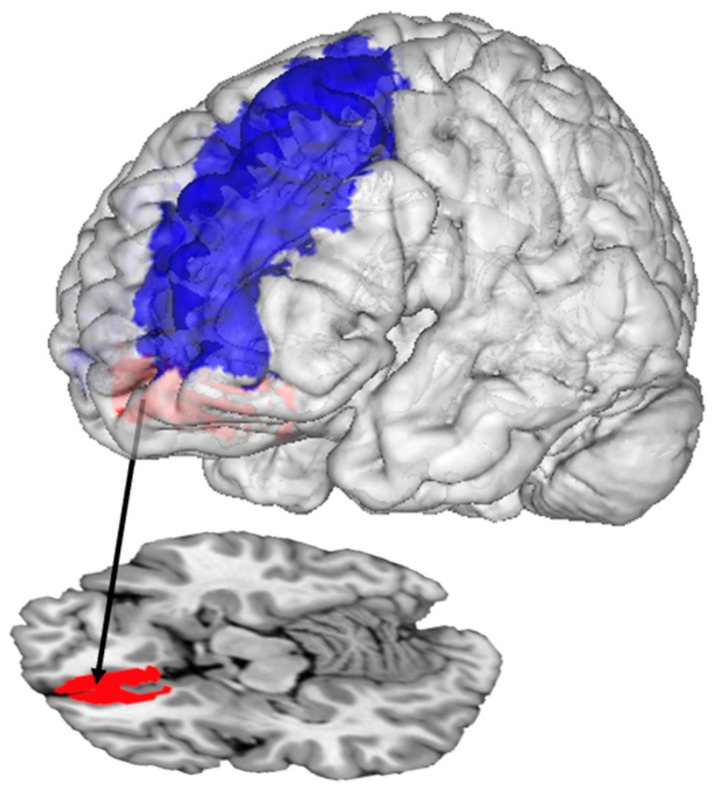
The middle frontal gyrus (blue) and medial frontal gyrus (red) had more activation during gum-chewing with full dental-arch dentures than shortened dental-arch dentures.

Instead of evaluating fully edentulous patients, Shoi et al. [25] recruited 11 patients with missing mandibular molars (1 male and 10 females, mean age ± SD: 66.1 ± 8.9 years, some mandibular premolars could be missing, and wearing old mandibular removable partial dentures for 1 month–8 years) and asked them to chew a stick of tasteless, odorless gum during fMRI scans. This was the only study in this review that investigated partially edentulous patients. The patients were given two new mandibular partial dentures, one replacing missing teeth until the second molar and one replacing missing teeth until the second premolar. In other words, one denture was standard, and the other had a shortened dental arch. The fMRI scans were performed twice: Two weeks after wearing each denture. Six of the patients wore the full dental-arch dentures first, whereas five wore the shortened dental-arch dentures first. The authors reported that patients had significantly higher activation in the bilateral medial and middle frontal gyri during gum-chewing when they had worn the full dental-arch dentures, relative to wearing the shortened dental-arch dentures. Simultaneously, the patients showed significantly better food comminuting and food mixing abilities, and higher subjective chewing ability perception with the full dental-arch dentures. The authors concluded that the medial and middle frontal gyri might be associated with masticatory function such as food comminuting and food mixing, which could be improved by having a dental arch fully replaced instead of partially replaced (Figure 4).

### 3.3. Rehabilitation Protocol and Brain Function

The aforementioned study by Padmanabhan et al. [24] evaluated the brain activation of the patients in response to memory recall tasks. The task was simple: the patients were given three random words half an hour before the fMRI scan, and then they would need to recall these words during the scan, as instructed. The results showed that patients had significantly higher brain activation in the hippocampus and the prefrontal cortex when they had been wearing implant-supported overdentures, compared to complete dentures. In addition, the patients had significantly higher Mini-Mental State Examination Test (MMSE) scores after they had worn implant-supported overdentures for 3 months (median score: 25.5), as compared to wearing complete dentures for 3 months (median score: 24.0), which was also better than being fully edentulous (median score: 20.5). However, readers should be aware of the limitations during the interpretation of the results of the MMSE. First, the sample size was small (*n* = 10) with a large age range (62–91). The authors did not elaborate on whether the MMSE score had accounted for the small sample size. Second, three Wilcoxon signed-rank tests were performed to compare the pair-wise differences in the MMSE scores of the patients among the fully edentulous periods, complete dentures, and implant-supported overdentures. There was no elaboration on whether the *p*-values had been adjusted for multiple analyses. Strictly speaking, if the test results had been considered significant if *p* < 0.017 (i.e., 0.05/3), then the differences between the complete dentures and implant-supported dentures would have been insignificant. The differences between fully edentulous and complete dentures would have become marginally significant (*p* = 0.017). Third, the authors did not re-test the patients by removing their dentures (returning them to a fully edentulous state). Therefore, the improved MMSE score could potentially be attributable to the patients becoming more familiar with the testing procedure upon repeated administration. Finally, a newer version of the MMSE known as the MMSE-2 was released in 2010 [35], which had good reliability [36] and could have been utilized instead of the old version. Whether the prolonged wearing of different dentures could affect the cognitive performances of patients should be investigated by future studies.

Meanwhile, the aforementioned study by Tan et al. [31] evaluated the brain activity levels in response to a continuous performance task aimed to assess the sustained attention and working memory of patients, as well as to a go/no-go task that assessed the impulsivity and inhibition. For the former task, a series of 120 yellow or white alphabet letters (B, C, D, or G) were presented to the patients in a predefined order. Patients needed to press a button when an identical yellow alphabet letter appeared twice consecutively. For the latter task, the patients were presented with the word “press” in green or red, and they needed to press a button when it was green (go) and refrain from pressing the button when it was red (no-go). The results indicated that both tasks showed reduced brain activity in the dorsolateral prefrontal cortex and posterior parietal cortex, one week after switching from complete dentures to implant-supported overdentures, but then returned to baseline levels six weeks after wearing implant-supported overdentures. It should be noted that results from this study were descriptive without statistical tests performed due to the small sample size (*n* = 4).

### 3.4. Uncharted Waters and Future Perspectives

The ultimate goal of replacing missing teeth is not only to restore the masticatory function but also to improve appetite and enhance nutrition. The aforementioned studies with full/partial fixed/removable denture rehabilitation with/without implant support did not evaluate the patients’ brain responses to visual or oral taste stimuli (food pictures and liquid food, respectively), a recurring research theme in the nutritional neuroscience community that usually includes criteria on subjects’ age, body-mass index, and presence/absence of eating disorders, but not the status of their dentition [18,37]. Another aspect would be any differences in their food choices between fixed versus removable denture rehabilitation, with or without implant support. Currently, virtual reality systems have been compatible with fMRI machines and allow subjects to navigate in a virtual supermarket and choose food items, as desired [38]. Apart from evaluating the brain activity levels of subjects performing tasks related to health considerations, self-control, and valuation [38], subjects could also have been evaluated to determine if they had regained their food preference, regardless of the food hardness. Moreover, a previous fMRI study reported numerous brain regions attributable to processing food hardness, including the supplementary motor area, the premotor area, and the prefrontal cortex [39]. Therefore, future gum-chewing studies could consider using gums with several hardness levels, and comparing patients with natural dentition and patients with various denture rehabilitation treatments, to assess if their brain activity levels would be comparable. Regardless of the use of an MRI scanner, patients with various fixed/removable denture rehabilitation with/without implant support should be asked to evaluate the perceived palatability, preference, and variations in food intake, as related to items and dishes. As compared to healthy controls with natural dentition, would these patients still prefer softer food or liquid food, even if their measurements on masticatory performance were not inferior? Or would they avoid fiber-rich food that could be easily trapped around or underneath the dentures, or sticky food that could loosen the dentures? These perceptions, preferences, and behaviors could ultimately affect the actual food intake and nutrition of the rehabilitated patients, but they must be elucidated in future studies.

Meanwhile, it should not be neglected that many neuropeptides and hormones regulate appetite, such as leptin, ghrelin, and orexin [12,40,41]. Any differences or changes in their levels upon prolonged wearing of different dentures should be evaluated, along with age and sex-matched controls with natural dentition. Moreover, neuropeptides and hormones usually modulate activities in the hypothalamus and other basal ganglia [41]. However, the findings from the current fMRI studies on fixed/removable denture rehabilitation with/without implant support focused on the activation in the cerebral cortex. Therefore, future fMRI studies should also consider evaluating the levels of these appetite-related neuropeptides and hormones and correlate them to the fMRI data.

The authors would like to make several recommendations for future studies, apart from the aforementioned research directions. First and foremost, more fMRI studies on fixed/removable denture rehabilitation with/without implant support should be encouraged, so that a meta-analysis of the existing findings can be performed. More research groups should be involved in this field, and they should apply more well-defined inclusion and exclusion criteria to better control the confounding factors, such as emotional factors, age, and general disease. Currently, it is recommended that a meta-analysis on neuroimaging studies should be composed of at least 17–20 studies to obtain robust results [42,43,44]. Even if this recommendation were neglected, the data reported in the nine original studies reviewed here would not constitute a neuroimaging meta-analysis, which generally requires brain coordinates of activated regions to be reported based on whole-brain analysis. Unfortunately, most of the studies only reported the names of the activated brain regions, without the coordinates, such as [24,27,28,29,31,32]. Consequently, the brain coordinates could only be identified in three studies: complete denture > implant-supported overdenture during chewing [30]; lower removable partial dentures with full dental arch > shortened dental arch during chewing [25]; and new complete dentures > old complete dentures during chewing [23]. Therefore, future studies should conduct standard whole-brain analyses on the fMRI data to report the brain coordinates of the activated regions, as opposed to only reporting the names of the brain structures or subjectively determining the strength of the fMRI signals based on visual inspection. For each original study, the sample size should be considered carefully. It is understandable that dental implant placement is a form of surgery, so it may not be easy to recruit a large cohort. In reality, the sample sizes of these denture studies were comparable to similar fMRI studies that recruited fully dentate subjects for gum-chewing and clenching (*n* = 10–20, refer to a recent meta-analysis [45]). However, this number was relatively small, as the median sample size of taste and food fMRI studies published in the 2010s was 35 [37].

## 4. Conclusions

Overall, this review covered nine fMRI studies on denture rehabilitation. Eight of them recruited completely edentulous patients, whereas one recruited partially edentulous patients. Seven studies compared within-group differences in the brain responses to various oral and cognitive tasks pre- and post-intervention (or intervention A versus intervention B), whereas two studies compared between-group differences, i.e., two groups received different interventions. The denture rehabilitation options involved in the studies were complete dentures (both arches), implant-supported overdentures (usually implied maxillary complete dentures with mandibular implant-supported overdentures), and implant-supported fixed dentures (both arches). The risk-of-bias assessments revealed some concerns in all nine studies, mostly regarding with blinding of the outcome assessments. Four were found to have a high risk of bias regarding either the blinding of the subjects or the incomplete outcome/data reporting. Jaw-clenching was the most frequently investigated task, followed by gum-chewing. Other tasks were evaluated by fMRI, one study each, such as teeth-tapping, lip-pursing, memory recall, continuous performance tasks, and go/no-go tasks. The evidence from the jaw-clenching studies suggested that the implant-supported fixed dentures appeared to be the best option, as compared to implant-supported overdentures and complete dentures, as it was associated with higher brain activity levels in various brain regions, including those corresponding to the primary sensory and motor cortices. Meanwhile, replacing old complete dentures with new ones could require a habituation process of about three months, after which the brain activity levels in response to jaw-clenching would return to baseline levels. Furthermore, the evidence from the gum-chewing studies showed that complete dentures had led to higher brain activity than the implant-supported overdentures. It was suggested that complete dentures were usually more mobile and induced a “more kinematically irregular and unstable chewing pattern” than implant-supported overdentures, thus triggering more brain activation. In addition, the studies found that the medial and middle frontal gyri could be associated with masticatory functions such as food comminuting and food mixing, which could be improved by having a dental arch fully replaced instead of partially replaced. In addition, wearing complete dentures of better quality (new versus old) could improve the masseter muscle function and chewing ability, which could also be beneficial for attention and memory functions. Preliminary yet circumstantial evidence from a memory recall study found that patients had significantly higher brain activation in the hippocampus and the prefrontal cortex after they had worn implant-supported overdentures for three months, versus wearing complete dentures for three months. All the fMRI studies demonstrated the functional neuroplasticity of patients undergoing denture rehabilitation, and they suggested that some rehabilitation options were more beneficial in restoring the masticatory functions and associated brain-activity levels.

## Figures and Tables

**Table 1:** Summary of the nine included studies.

Study	Edentulism	Results Comparison	Study Site	Participants	Mean Age in Years ± SD (Range)	Denture Rehabilitation Sequence	Task during fMRI Scan	Evaluation Timing	Conclusions from fMRI Results
Yan et al., 2008 [27]	Complete	Between-group	China	20 (13M, 7F): 8 CD (5M, 3F), 9 IOD (5M, 4F), 3 IFD (3M)	59.9 ± 7.4. CD: 61.5 ± 8.1. IOD: 59.1 ± 8.3. IFD: 58.0 ± 2.6	CD, IOD, or IFD. IOD group: either IOD for both arches, or CD/Mx + IOD/Md. Implant no. for IOD/IFD: 0–12/Mx and 4–12/Md	Clenching	With dentures (worn for 8 months–5 years 8 months)	higher proportion of IFD patients had brain activation than IOD and CD patients
Kimoto et al., 2011 [30]	Complete	Within-group	Japan	4 (3M, 1F)	(64–79)	CD, then IOD. IOD: CD/Mx + IOD/Md. Implant no. for IOD: 2/Md (standard Brånemark-system protocol)	Gum-chewing	1 month after wearing CD, and 1 month after wearing IOD	brain activation with CD > IOD
Luraschi et al., 2013 [29]	Complete	Within-group	Australia	11 (6M, 5F)	71.4 ± 4.8	CD	Clenching, denture teeth-tapping, and lip-pursing	Wearing old CD, right after receiving new CD, 1 month and 3 months after wearing new CD	Clenching: brain activation at 1 month with new CD > old CD, but back to normal level at 3 months with new CD. Tapping and lip-pursing: no temporal change in brain activation
Shoi et al., 2014 [25]	Partial	Within-group	Japan	11 (1M, 10F)	66.1 ± 8.9	Lower RPD with full dental arch, then RPD with shortened dental arch (up to 2nd premolar). Or vice versa (2 randomized crossover groups)	Gum-chewing	2 weeks after wearing each of the lower RPD	Brain activation with full dental arch RPD > shortened dental arch RPD
Tan et al., 2020 [31]	Complete	Within-group	Australia	4 (2M, 2F)	73.0 ± 1.4	CD, then IOD. IOD: CD/Mx + IOD/Md. Implant no. for IOD: 2/Md	Clenching, continuous performance task, Go/No-Go task	3 months after wearing CD, 1 week after wearing IOD, and 6 weeks after wearing IOD	Brain activation at 1 week with IOD < CD, but back to normal level at 6 weeks with IOD
Nakasato et al., 2021 [23]	Complete	Within-group	Japan	14 (4M, 10F)	80.2 ± 5.9	CD	Gum-chewing	Wearing old CD, and 3 months after wearing new CD	Brain activation with new CD > old CD
Verma et al., 2021 [32]	Complete	Within-group	India	12 (gender undisclosed)	59.2 (40–70)	CD, then IOD. IOD: Details unclear. Likely to be CD/Mx + IOD/Md. Implant no. for IOD: 2/Md (standard Brånemark-system protocol)	Clenching	3 months after wearing CD, and 3 months after wearing IOD	Brain activation with IOD > CD
Padmanabhan et al., 2022 [24]	Complete	Within-group	India	10 (4M, 6F)	(62–91)	CD, then IOD. IOD: CD/Mx + IOD/Md. Implant no. for IOD: 2/Md	Gum-chewing, and memory recall	Before treatment, 3 months after wearing CD, and 3 months after wearing IOD	Gum-chewing: brain activation with IOD < edentulous; IOD vs CD—mixed results Memory recall: brain activation with IOD > CD MMSE score (median): before treatment (20.5) < complete denture (24.0) < IOD (25.5)
Bhattacharjee et al., 2022 [28]	Complete	Between-group	India	18 (10M, 8F): 6 CD (2M, 4F), 6 IOD (4M, 2F), 6 IFD (4M, 2F)	60.7 ± 4.0. CD: 61.0 ± 4.7. IOD: 59.8 ± 4.0. IFD: 61.3 ± 3.7	CD, IOD, or IFD. IOD group: CD/Mx + IOD/Md. IFD group: IFD for both arches. Implant no. for IOD: 2/Md, and for IFD: all-on-4 or on a case-by-case basis	Clenching	With dentures	Higher proportion of IFD patients had brain activation than IOD and CD patients

CD, complete denture. IFD, implant-supported fixed denture. IOD, implant-supported overdenture. Md, mandible. MMSE, Mini-Mental State Examination. Mx, maxilla. RPD, removable partial denture.

## Data Availability

Not applicable.
